# Editorial: Physical education, health and education innovation, volume II

**DOI:** 10.3389/fpsyg.2025.1621248

**Published:** 2025-05-30

**Authors:** Paulo Jorge Martins, David Manzano-Sánchez, Noelia Belando-Pedreño, Jorge Carlos-Vivas, Manuel Gómez-López

**Affiliations:** ^1^Faculty of Human Motricity, University of Lisbon, Lisbon, Portugal; ^2^Faculty of Education and Psychology, University of Extremadura, Badajoz, Spain; ^3^Faculty of Sports Sciences, European University of Madrid, Madrid, Spain; ^4^Faculty of Sports Sciences, University of Murcia, Murcia, Spain

**Keywords:** physical education, healthy habits, physical activity, mental health, teacher training, educational technology, youth, inclusion

Contemporary society demands the training of children and adolescents in environments that promote respect and educational values. That being said, educating children and adolescents extends beyond the mere transmission of academic content. It involves fostering individuals who are healthy, responsible, and prepared to face life’s challenges. This requires an educational environment grounded in respect, human values, and practices that promote physical, mental, and social well-being. In this context, Physical Education (PE) emerges as a pivotal discipline in promoting healthy habits, encompassing regular physical activity, proper nutrition, and the reduction of sedentary behavior.

The World Health Organization (WHO) warns of the high rates of overweight and obesity, urging immediate action. In fact, the WHO has highlighted the concerning rates of overweight and obesity among young people in various countries, emphasizing the need for preventive actions starting from the early stages of schooling. Formal education, especially Physical Education, plays a crucial role in improving healthy habits through educational programs. Since the 20th century, studies have increased in educational centers and sports schools, not only promoting physical activity but also seeking to generate long-term adherence among students and athletes. This second volume of the Research Topic *Physical Education, Health and Education Innovation* includes contributions that highlight innovative programs promoting healthy habits related to physical activity, nutrition, and mental well-being in formal education, including primary, secondary, and university students. As said, schools, as significant social institutions, play a leading role in combating these issues, particularly through educational programs integrated into PE classes. Recent research indicates that as students progress through the educational system, especially in Secondary Education, there is a tendency to decrease physical activity and increase behaviors detrimental to health, such as alcohol and tobacco use, poor nutrition, and sedentary lifestyles. Therefore, PE should not be limited to encouraging occasional sports practice but should aim to develop continuous adherence to physical activity and sport throughout students' lives. Innovative interventions, such as that proposed by Deng et al., involving complex exercises like fancy rope-skipping, have demonstrated benefits not only in motor coordination but also in developing cognitive skills like selective attention and concentration. Incorporating such practices into the school curriculum can be an effective and accessible strategy to enhance students' holistic development.

Another critical aspect related to physical activity is its contribution to mental health and social interaction within the school environment. Research by Galán-Arroyo et al., shows that physical self-efficacy—the perception of one's motor competence—can act as a protective factor against bullying. Students who feel physically capable tend to exhibit greater self-confidence and social skills, reducing their vulnerability to situations of violence or exclusion.

In this regard, PE also becomes a privileged space for developing pro-social values such as empathy, cooperation, and respect for diversity. Ongoing teacher training, as discussed by Hernández-González et al., should include inclusive pedagogical strategies capable of meeting the needs of all students, including students with disabilities, fostering an inclusive and equitable school environment.

Interventions conducted in Primary Education, such as the study by Gallotta et al., reinforce the importance of balancing study time with moments of movement and physical stimulation. Simple and playful activities, like rope skipping, have shown positive effects on motor coordination and attention in children aged 7–9 years. Similarly, Urbano-Mairena et al., highlight the benefits of active breaks during lessons, which can significantly increase physical activity levels without compromising academic performance. Moreover, a positive effect has also been reported as a result of approaches that combine sport and learning to promote the healthy development of adolescents with intellectual disabilities. The study by Wang et al., shows that a Badminton ‘Body-Smart Integration’ intervention can improve the cognitive ability of students with intellectual disabilities, with the latter showing a significant improvement in cognitive ability compared to students in the badminton group and the control group.

Beyond childhood, the implementation of physical activity programs has also proven effective in reducing screen time among university students, as observed by Özkara et al. Encouraging participation in active leisure activities contributes to a healthier and more balanced routine. Although often seen as a culprit in promoting sedentary behavior, technology, when well applied, can be an ally in the educational process. Cui et al., identified a positive relationship between the use of digital technologies and students' physical outcomes, provided there is genuine engagement with the proposed activities. In this case, engagement serves as a key mediator for the success of technological interventions.

Zhao and Ji, on the other hand, explored the use of virtual reality in teaching sailing and showed that this technology can significantly enhance students' intrinsic motivation and learning compared to traditional methods. This approach appears promising, especially in contexts where practical teaching faces structural limitations.

In the sports field, the coach plays a central role in the development of young athletes. Studies like that of Liu et al., reveal that individualized strategies, combining physical and psychological aspects, are essential for guiding athletes toward healthy and sustainable choices.

However, the competitive environment can sometimes favour a culture of harmful perfectionism. Meng et al., discuss how this personality trait, coupled with intense external demands, can jeopardize athletes' performance and well-being. However, when well mediated, this same climate can be transformed into a positive challenge. It is up to the coach to promote a motivational environment that values effort, personal achievement and teamwork, helping to strengthen the athletes' psychological capital and self-efficacy. Finally, promoting healthy habits and an active lifestyle should be a central priority in educational policies. Physical Education, when integrated into an innovative, inclusive pedagogical proposal based on scientific evidence, proves to be a powerful ally in the formation of autonomous, healthy individuals who are prepared for life in society. It is essential that schools, families, communities and public policy makers work together to build educational programmes that combine physical activity, nutrition and mental well-being. Only with an integrated and committed approach will it be possible to reverse the current trends of sedentary lifestyles and illness among children and adolescents. As we look to the future of education, it is clear that the health and well-being of students must be placed at the centre in the classroom—not as peripheral issues, but as the foundations for academic success and for building a more balanced and just society.

